# Associations of the Mediterranean-Style Dietary Pattern Score with Coronary Artery Calcification and Pericardial Adiposity in a Sample of US Adults

**DOI:** 10.3390/nu14163385

**Published:** 2022-08-18

**Authors:** Leigh Ann Richardson, Arpita Basu, Lung-Chang Chien, Amy C. Alman, Janet K. Snell-Bergeon

**Affiliations:** 1 Department of Epidemiology and Biostatistics, University of Nevada at Las Vegas, Las Vegas, NV 89154, USA; 2 Department of Kinesiology and Nutrition Sciences, University of Nevada at Las Vegas, Las Vegas, NV 89154, USA; 3 College of Public Health, University of South Florida, Tampa, FL 33612, USA; 4 Barbara Davis Center for Childhood Diabetes, Anschutz Medical Campus, University of Colorado, Aurora, CO 80045, USA

**Keywords:** coronary artery calcification, pericardial adiposity, Mediterranean-Style Dietary Pattern Score

## Abstract

Several studies have identified improvements in the risks of cardiovascular disease in adults following a Mediterranean dietary pattern. However, data are scarce on its association with coronary artery calcification (CAC) and pericardial adiposity (PAT) in US adults with and without diabetes. To address this gap, we conducted a case-control study using baseline data from the Coronary Artery Calcification in Type 1 Diabetes (CACTI) study [*n* = 1255; Type 1 Diabetes (T1D): *n* = 563; non-Diabetes Mellitus (non-DM): *n* = 692]. Participants completed a validated food frequency questionnaire, fasting (12 h overnight fast) biochemical analyses, and a physical examination including anthropometric measures. CAC and PAT were measured using electron beam-computed tomography. Logistic regression models were used to examine the associations of the Mediterranean-Style Dietary Pattern Score (MSDPS) with CAC (presence or absence), and linear regression models were applied to PAT analyses. In all of the adjusted models, no significant associations with CAC were found. For PAT, an increasing MSDPS was consistently associated with its lower volume in models adjusted for age, sex, diabetes status, total calories, and body mass index (all *p* < 0.05). The association between MSDPS and PAT was attenuated after adjusting for serum lipids and physical activity. In conclusion, the baseline data from the CACTI study show that a greater adherence to MSDPS is associated with a lower PAT volume and provide evidence that the Mediterranean dietary pattern is associated with lower cardiovascular risk markers.

## 1. Introduction

Cardiovascular disease (CVD) represents a collection of disorders associated with the heart and blood vessels and includes coronary artery calcification (CAC). The risks of these conditions can be increased by pericardial adiposity (PAT). CAC and PAT can serve as prognostic indicators for CVD [1,2]. CAC is associated with atherosclerosis (the thickening and hardening of arteries due to plaque build-up), and the mechanisms by which it forms include the loss of inhibitors of mineralization and the induction of osteogenesis [3]. CAC has been identified as having a moderate prognostic accuracy when compared to other traditional risk factors for diagnosing atherosclerotic CVD and predicting coronary heart disease (CHD)-related events, including mortality [4,5,6,7]. PAT is the sum of fat deposits found in the heart’s epicardial and pericardial regions [8]. In several studies, a higher volume of pericardial fat was associated with impaired diastolic dysfunction in otherwise healthy adults. PAT has been shown to impact the structure, function, and outcome of the left ventricle independent of insulin resistance, inflammation, and hepatic fat, leading to a higher all-cause mortality rate in older adults [9,10,11]. Additionally, PAT was associated with a poor CVD prognosis, including non-fatal and fatal myocardial infarctions, sudden cardiac arrest, heart failure, angina, stroke, and death [11].

In individuals with diabetes, CVD is the leading cause of death, with heart disease and stroke being twice as likely for these individuals compared to those without diabetes [12]. Adults with diabetes also have a higher risk of CAC, and those who suffer from chronic kidney disease are at an even higher risk [13,14]. It has been estimated that, for every 1% increase in HbA1c, there is an approximately 11–16% increase in CVD events [15]. In a separate study, the relative risk of CHD was 2.37 (95% CI: 1.50–3.72) when comparing the highest to the lowest quintile of HbA1c, which accounted for additional CHD risks [16]. Prior to the Diabetes Control and Complications Trial (DCCT)/Epidemiology of Diabetes Interventions and Complications (EDIC) cohort, CAC was identified as a predictive indicator for CVD events in the general population and in individuals with T2D, but not in those with T1D [17]. However, researchers demonstrated that CAC had a similar prognostic effect for CVD risk in individuals with T1D [17]. Additionally, adults with diabetes have been associated with having higher volumes of PAT than those without diabetes, and this relationship is independent of BMI and visceral adipose tissue [18]. Compared to normoglycemic individuals, adults with pre-diabetes and diabetes were also found to have a greater volume of PAT [19]. Lastly, adults with T1D were associated with increased cardiac adipose tissue and subclinical atherosclerosis due to alterations in high-density lipoprotein composition, and youth with T1D also reported higher cardiac adipose tissue than controls [20,21].

While a healthy diet remains the cornerstone in CVD prevention and management, this strategy has manifold definitions. The American Diabetes Association recommends that adults with diabetes consume diets high in plant-based foods, lean proteins, and fiber [22]. The Center for Disease Control and Prevention recommends a diet high in fruits, vegetables, and fiber and low in saturated fats, trans fats, and cholesterol [23]. Both agencies utilize different approaches to advising individuals to eat a healthier diet. The ADA uses a plate approach to help an individual visualize how much of their plate should be dedicated to certain food groups, and the CDC highlights how certain components such as excess salt and saturated and trans fats can be harmful and contribute to heart disease. While these approaches are helpful and align with current knowledge about different dietary components, they are not as exact as a dietary pattern, which includes specific serving sizes and frequencies of dietary components. Dietary patterns provide multiple benefits, which include a priori scoring, consistency in measurement, and applicability across different study populations.

Similar to the recommendations from the ADA and CDC, the Mediterranean Diet (MedDiet) is characterized as the increased consumption of fruits, vegetables, and whole grains and reduced quantities of saturated and trans fats. However, the major distinction is that the MedDiet provides specific serving sizes for each major component and has been utilized in many different study populations. The MedDiet was first identified for its cardiovascular benefits by Ancel Keys [24]. Since this finding, researchers have focused on further examining the association between the MedDiet and risks for CVD, including abdominal obesity, high-density lipoprotein (HDL), triglycerides, diabetes, and blood pressure [25,26,27,28,29,30]. To examine different levels of adherence to the MedDiet, dietary pattern scores have been used as an index of diet quality.

The MedDiet is characterized by a high habitual intake of vegetables, fruits, legumes, nuts, cereals (primarily whole grains), and olive oil and a low-to-moderate consumption of dairy, fish, meat, and wine [31,32,33]. However, applying a MedDiet dietary pattern score has not been consistent in the literature [33,34]. The original MedDiet, based on the habitual eating patterns of the Mediterranean populations, has been adapted to different non-Mediterranean populations, resulting in different ways to calculate the dietary pattern scores [34,35]. One of the first MedDiet scores was developed by Trichopoulos et al. to assess the MedDiet and overall survival in a Greek population [36]. The Trichopoulos et al. score was calculated based on sex-specific median values for the following eight components: monosaturated:saturated fat ratio, ethanol consumption, legumes, cereals (including bread and potatoes), fruits, vegetables, meat and meat products, and milk and dairy [36]. The score ranged from 0–8. To receive a point for each food category, individuals had to consume at or above the median for the monosaturated:saturated fat ratio, legumes, cereals, fruits, and vegetables; consume less than the median for red meat and dairy products; and consume moderate amounts of ethanol (excluding heavy drinkers) [36]. In a later study by Trichopoulou that added fish consumption to the calculation (9-point scale), approximately 31% of the population had low adherence (0–3 points), 43% had moderate adherence (4–5 points), and 26% had high adherence (6–9 points) to a MedDiet [32]. Rumawas et al. created a Mediterranean-Style Dietary Pattern Score (MSDPS) to assess how non-Mediterranean populations adhere to a MedDiet pattern [35]. The scoring of the MSDPS establishes recommended intake levels for each dietary component. Points are deducted for overconsumption (consuming beyond the recommended intake level). Additionally, the score is also adjusted based on the dietary components that do not adhere to a MedDiet, such as beer, hotdogs, and white bread typically consumed by the US population. The quintile scores for adherence to this method ranged from 3.0–17.8, 17.9–22.6, 22.7–26.8, 26.9–31.6, and 31.7–60.7 [35]. The overall MSDPS represents how well individuals meet recommended quantities for each component and how well their diet aligns with a MedDiet.

While several studies have assessed diet quality using the MedDiet adherence in relation to CVD risks, few studies have assessed the associations of a MedDiet with CAC and PAT outcomes in individuals with and without diabetes. A few observational studies have applied MedDiet scores to assess CAC and other coronary plaque measures in populations that included individuals with diabetes and reported an inverse association [37,38]. Additionally, clinical studies have reported the effects of a MedDiet intervention in decreasing plaque and increasing flow-mediated dilation in adults [39,40,41]. Keeping this gap in the literature in view, we aimed to examine if diet quality based on the MedDiet is associated with the prevalence of CAC and PAT in the Coronary Artery Calcification in Type 1 Diabetes (CACTI) cohort, including adults with and without type 1 diabetes (T1D).

## 2. Methods

### 2.1. Participants

For this report, baseline data (2000–2002) from the CACTI study were used. The primary objective of the CACTI study was to examine the progression of subclinical atherosclerosis and its risks in individuals with a T1D diagnosis and in a group of non-diabetic controls, all of whom were free from CVD at enrollment in a case-control design [42]. The healthy non-diabetic controls were recruited among spouses, friends, or neighbors of the T1D participants and had to have a fasting blood glucose < 126 mg/dL. The T1D participants were insulin-dependent within a year of diagnosis and were diagnosed before the age of 30 or had positive antibodies or a clinical course consistent with T1D. The CACTI study had 1416 individuals between 19 and 56 years old with no known history of CVD: 652 T1D participants and 764 control participants. The study was approved by the Colorado Multiple Institutional Review Board, and all participants provided informed consent. The study was registered at clinicaltrials.gov (NCT00005754, accessed on 17 August 2022).

### 2.2. Dietary Intakes and MSDPS Calculations

The participants completed a 126-item, validated semi-quantitative food frequency questionnaire (Harvard FFQ, 1988) [43]. The USDA food composition database was used to analyze the nutritional composition of the foods in the FFQ, which has been previously described [43]. The FFQ collected the frequency of food consumption for each food item during the past 12 months (frequency options: ‘never or less than once per month’, ‘1–3 times per month’, ‘1 per week’, ‘2–4 per week’, ‘5–6 per week’, ‘1 per day’, ‘2–3 per day’, ‘4–5 per day’, and ‘6+ per day’). Standard portion sizes were provided in natural units (e.g., a slice of bread, one apple, one egg, one can of soda) or in household measures (e.g., cup, ounce, teaspoon). Daily food consumption was calculated by multiplying the frequency of food consumption by the standard portion sizes. A total of 1306 participants completed the FFQ [44]; however, individuals were further excluded using Willett’s criteria, which have been previously described as having extremely high or low caloric intakes and the removal of missing values (*n* = 11) [45]. With the exclusion criteria applied, 692 non-diabetic controls and 563 T1D participants were included in this case-control analysis [44,45].

To calculate a MedDiet score, the Mediterranean-Style Dietary Pattern Score (MSDPS) was applied. The MSDPS was developed by Rumawas et al. to assess conformity to the Mediterranean diet pyramid [35]. In short, the MSDPS was developed to assess adherence to the MedDiet, which is characterized by following the appropriate servings per day or servings per week for each food group, with a penalty for overconsumption. The scoring system includes a proportional weight to identify foods that align with the MedDiet. Twelve of the thirteen components (whole grains, fruits, vegetables, dairy, wine, fish, poultry, legumes, potatoes, eggs, sweets, meat) are measured on a continuous scale with a maximum score of 10 (complete adherence to MedDiet) and a minimum score of 0 (lack of adherence to MedDiet). Olive oil was scored as follows: 0 for no use, 5 for olive oil use with other vegetable oils, and 10 for the use of olive oil only. The sum of all 13 component points equates to 130 total points, which is then multiplied by 100 to provide an unadjusted percentage. The final step is multiplying the unadjusted percentage by the total energy intake from the MedDiet food groups. The Framingham Heart Study utilizing MSDPS identified scores of 4–19 as low adherence, scores of 19.1–25.0 as moderate, and scores of 25.1–50.9 as high adherence in their population [46]. In the Framingham Heart Study Offspring Cohort, quintile scores ranged from 4.05–17.7, 17.9–21.6, 21.7–25.0, 25.1–29.0, and 29.1–49.6 [47].

### 2.3. Study Measurements

Demographics, medical history, physical activity level (Modifiable Activity Questionnaire), and medication use were collected from standardized questionnaires for all participants, as previously described [48]. Height, weight, waist and hip circumference, and systolic and diastolic blood pressures were collected via a physical examination. Blood pressure measurements, resting systolic blood pressure (SBP), and fifth-phase diastolic blood pressure (DBP) were taken in triplicate while the subject was seated, following a 5 min rest. The average of the second and third readings was used for the study. Individuals with an SBP greater than or equal to 140 mmHg or a DBP greater than or equal to 90 mmHg or who took antihypertensive medicines were categorized as hypertensive. Total cholesterol, high-density lipoprotein (HDL), and triglycerides were measured using a fasting blood sample, which was stored at −80 °C until assayed. The Friedewald equation was used to calculate low-density lipoprotein (LDL). Glycated hemoglobin (HbA1c) was measured using high-performance liquid chromatography (HPLC, BioRad variant, Hercules, CA, USA).

### 2.4. Measurement of CAC

To determine CAC, two measurements were collected using an ultrafast Imatron C-150XLP electron beam computed tomography (EBCT) scanner and averaged (Imatron, South San Francisco, CA, USA) [48]. The presence of CAC was annotated as CAC > 0 on either scan and indicated any amount of CAC.

### 2.5. PAT Volume Measurement

Analyze Direct 11.0 volume analysis software was used to manually trace the heart using a spline edge detection feature, and boundaries included the chest wall, descending aorta, and bronchus. The PAT volume assessment started 3 mm above the left main coronary artery and was repeated for each 3 mm slice until 30 mm below the left main coronary artery [44]. The PAT volume was then computed using thresholds of −190 to −30 Hounsfield units, which were used to differentiate fat from other tissues [44]. The PAT volume included internal and external pericardium adipose tissue.

### 2.6. Statistical Analysis

The main objective of the present study is to examine the associations of the MSDPS with CAC and PAT prevalence at the baseline of the CACTI cohort. To measure differences between the control group and the T1D group, an independent-samples t-test was conducted to analyze differences in risk factors, MSDPS, and individual components of the MSDPS. The Wilcoxon rank sum test was used to compare differences in continuous variables with skewed distributions. Baseline comparisons for sex, race (non-Hispanic white (NHW)), Hispanic origin, and CAC measurements as categorical variables between two groups were analyzed using chi-square tests.

The logistic regression model examined the association of MSDPS with CAC (presence or absence). The application of the logistic regression model provides an odds ratio for the parameter estimate, which can give the change in odds of CAC for a one-point increase in the MSDPS. For the CAC outcome, the models were adjusted for appropriate confounding variables as listed: model 1 (age, sex, and diabetes status), model 2 (model 1 + total calories + HbA1c + Body Mass Index (BMI)), and model 3 (model 2 + HDL + LDL+ systolic blood pressure).

Linear regression models were applied to the PAT analyses. The application of the linear regression model provides a parameter estimate to indicate the volume change per one point increase in the MSDPS. For the PAT analyses, the PAT volume was log-transformed to meet equal variance and normality assumptions. The models applied to investigate the associations between MSDPS and PAT volume were adjusted as follows: model 1 (age, sex, and diabetes status), model 2 (model 1 + HbA1c + total calories), model 3 (model 1 + BMI), and model 4 (model 1 + LDL + triglycerides + physical activity). In an exploratory analysis, race and Hispanic origin were added to Model 1 in CAC and PAT analyses, and lipid-lowering medications were adjusted in the final models for CAC and PAT analyses.

Additionally, an interaction analysis was conducted between the MSDPS and diabetes status, and no significant interaction was found. Thus, the analyses were adjusted for diabetes status using the pooled sample. We also present the data stratified by diabetes status for comparisons. The statistical analyses were performed using SAS v9.4 (SAS Institute, Cary, NC, USA). A two-sided alpha level of 0.05 was used to define statistical significance.

## 3. Results

Table 1 shows the baseline characteristic differences between the control and T1D participants. Overall, the non-diabetic control group was significantly older and had a greater percentage of females and Hispanic participants, a higher LDL, higher triglycerides, and a higher PAT than the T1D group. In contrast, the T1D participants were predominately non-Hispanic whites (NHW), had a significantly higher HDL and systolic blood pressure, and had more individuals with a CAC > 0 than the control group. The total caloric intake, level of physical activity, and BMI did not differ between the two groups. The BMI category allocation for participants without diabetes included 8 (1.1%) underweight (BMI < 18.5), 323 (46.4%) healthy (BMI between 18.5 and 24.9), 225 (32.3%) overweight (BMI between 25.0–29.9), and 139 (19.9%) obese (BMI greater than or equal to 30.0) participants. The BMI category allocation for participants with T1D included 3 (0.5%) underweight, 244 (42.8%) healthy, 230 (40.4%) overweight, and 88 (15.47%) obese participants.

In Table 2, the CAC measurements showed nonsignificant associations with the MSDPS score in the pooled analysis as well as in the analysis stratified by diabetes. As reported in Table 3, after adjustment for age, sex, diabetes status, total calories, and BMI, we found a significant association between the MSDPS and PAT in the pooled analysis, where, for every one-point increase in the MSDPS, there was a significant −0.003 cm^3^ (95% CI: −0.006, −0.0004; *p*-value = 0.025) decrease in PAT volume. Further adjustment for LDL, triglycerides, and physical activity, however, attenuated the association between the MSDPS and PAT volume in the pooled analysis. When stratified by diabetes status, the MSDPS and PAT volume revealed significant inverse associations in all of the adjusted models in the non-diabetic controls. No significant association was observed in the T1D group. These associations were not affected when further adjusted for race, Hispanic origin, and lipid-lowering medications.

In Table 1 and Table 4, the individual component scores of the MedDiet were analyzed for differences between the two groups and for their associations with CAC and PAT, respectively. In Table 1, the MSDPS-based whole grain score was significantly higher in the T1D group, but the wine and fish scores were significantly higher in the non-diabetic control group. After adjustment for age, sex, diabetes status, and total calories, Table 4 showed that none of the individual food components were significantly associated with a CAC > 0. For the PAT analyses, fruit, wine, and meat consumption were significantly associated with −0.02 cm^3^ (95% CI: −0.03, −0.01; *p*-value < 0.0001), −0.03 cm^3^ (95% CI: −0.05, −0.02; *p*-value = 0.0001), and −0.03 cm^3^ (95% CI: −0.04, −0.01; *p*-value < 0.0001) lower PAT, respectively.

## 4. Discussion

We observed an inverse association of increasing adherence to the Mediterranean-Style Dietary Pattern Score with pericardial adiposity in the models adjusted for age, sex, diabetes status, total calories, and BMI in pooled analyses; when LDL, triglycerides, and physical activity were added, this association was attenuated, indicating that the association was not independent of the roles of serum lipids and physical activity in cardiac adiposity. When stratified by the disease status, the association between adherence to the Mediterranean diet and PAT revealed significant inverse associations in non-diabetic controls only in all of the adjusted models. Adherence to the Mediterranean-Style Dietary Pattern Score was not significantly associated with the presence of CAC. When analyzing the individual scores of the MSDPS foods and associations with CAC and PAT, fruit, wine, and meat groups had a significant inverse correlation with PAT. The attenuation of the results in the model adjusted for lipids and physical activity is expected, since PAT has been significantly associated with LDL cholesterol, triglycerides, and physical activity [49,50,51,52].

Longitudinal studies have reported that increased adherence to the MedDiet is associated with reduced pericardial fat in the Framingham Heart Study and in the Multi-Ethnic Study of Atherosclerosis [53,54]. However, these studies did not include individuals with T1D and utilized different calculations for MedDiet scores. Some concerns with the previously reported MedDiet scores are that individuals have higher scores simply because they consumed more, and those scores do not account for foods that do not align with the MedDiet pattern. The intention of this study was to measure adherence to the MedDiet, which includes consuming the appropriate quantities of each food group, as recommended by the pattern, as well as accounting for overconsumption.

The inverse association between the MedDiet and pericardial adiposity in the pooled analyses is likely due to its known effects in reducing fat accumulation [55,56,57]. The MedDiet is commonly known for the 13 components described to create the MSDPS; other characteristics of the diet include low saturated and high monounsaturated fats, fiber, and antioxidants, which have protective effects by reducing lipids [58,59]. It is likely that these lipid-lowering dietary factors are associated with reduced fat accumulation in the pericardium. Interestingly, this inverse association was observed only in non-diabetic controls but not in adults with T1D. This could be explained by the significantly higher baseline PAT volume in the non-diabetic controls when compared to the T1D group in our cohort. Increased adherence to a Mediterranean diet has been reported to be associated with lower visceral adiposity in a cross-sectional study of adults without diabetes [60]. In another weight loss study, the Mediterranean diet was also shown to decrease intrapericardial adiposity in sedentary adults without diabetes [55]. However, such studies are lacking for T1D adults and warrant further investigation involving Mediterranean diet exposure or intervention in reducing pericardial fat volume.

When the individual MSDPS components were analyzed for association with PAT, the significant findings for fruit and wine scores conform to the findings in the literature, which indicate reduced CVD risk [61,62,63,64]. Animal studies have investigated the effects of fruits and their extracts, such as polyphenols and specific flavonoids, on adipose tissue inflammation and lipids. These studies show that fruit and fruit extracts protect against adipose tissue inflammation, lower the expressions of proinflammatory cytokines such as tumor necrosis factor-alpha (TNF-α), interleukin-6 (IL-6), and monocyte chemotactic protein-1 (MCP-1), reduce lipid synthesis, inhibit adipogenesis, control weight gain and adipocyte size, and lower serum total cholesterol and serum LDL-cholesterol [65,66,67,68,69]. Red wine is rich in resveratrol, which has been found to increase adipocyte apoptosis, fat mobilization, fatty acid oxidation, and lipolysis and decrease adipogenesis and lipogenesis [70,71,72]. Ultimately, resveratrol’s modulation of signaling pathways results in a decrease in adipocyte quantity and lipid accumulation [70]. Therefore, it is likely that these compounds in fruits and wine were associated with lower pericardial fat volume in our study.

The inverse association with the meat score was unexpected. The meat score reflects the level of adherence versus the overall meat consumption. To receive maximum points (10 points) for meat consumption, individuals would have to consume only one serving per week. Individuals who consumed less than one serving or greater than one serving per week received a lower score. For example, individuals who ate 0.5 servings of meat per week and those who ate 1.5 servings per week received the same score (5 points). Therefore, this score indicates that a higher adherence to the recommended level of meat consumption is associated with a lower PAT volume. Similarly, wine consumption, including white and red wine, assigned maximum points for modest consumption, meaning that adherence to the recommended levels was associated with a lower PAT volume.

When comparing our baseline MSDPS to the Framingham Offspring Cohort and the Atherosclerosis Risk in Communities (ARIC) cohort, the controls and T1D groups in our study showed similar scores of (mean ± SD) 25.4 ± 7.8 and 25.0 ± 7.8, respectively, compared to the score of 25.0 ± 8.21 for the Framingham cohort and the score of 20.0 ± 6.5 for the ARIC cohort [35,73]. Our study group had higher component scores for whole grains, dairy, wine, legumes, eggs, sweets, and meat when compared to the Framingham cohort and higher component scores for everything except fruits, fish, legumes, potatoes, sweets, and meat for the ARIC cohort [35,73]. Because the MSDPS calculation is relatively new, previous US cohort studies have utilized MedDiet scoring on different scales. One of the most common methods is the Alternate Mediterranean Diet score (aMED). This score calculates a sex-specific median score to assign one point to individuals who consume above the median for specific components (such as fruits, vegetables, and whole grains) or below the median for other components (such as meat and dairy products) [26]. The Reasons for Geographic and Racial Differences in Stroke (REGARDS) and the Nurses’ Health Study had mean aMed scores of 4.4 ± 1.7 and 4.3 ± 1.4, respectively [74,75,76]. Overall, our study sample of US adults revealed a low adherence to the MedDiet pattern.

Our insignificant findings for the association between MSDPS and the presence of CAC are contrary to previous reports, though not all of these studies examined CAC as a marker of atherosclerosis. A few longitudinal studies have shown that adherence to Mediterranean-style diets is associated with improved endothelial function and the limited progression of CAC [38,77,78]. The Heinz Nixdorf Risk Factors, Evaluation of Coronary Calcium and Lifestyle (RECALL) study was conducted in a German population of men and women between the ages of 45 and 75 years and measured CAC at baseline and at close to a 5-year follow-up. This study used a clustering technique for food groups but not the MSDPS or the aMED scores, and this may explain the overall difference in results [38]. In secondary analyses of the Coronary Diet Intervention with Olive Oil and Cardiovascular Prevention (CORDIOPREV) study conducted in Spain, researchers identified decreased intima-media thickness of common carotid arteries (IMT-CC) at 5 years, which was maintained at 7 years, and a higher endothelial function assessed by flow-mediated dilation (FMD) of the brachial artery, following MedDiet adherence [77,78]. Thus, these significant differences among the RECALL and CORDIOPREV cohorts and our study may be explained by differences in age, location, study design (mainly longitudinal vs. cross-sectional), and MedDiet assessment scale.

There are also a few other reasons explaining the insignificant association between MSDPS and the presence of CAC in our study. First, our study participants were not habitually consuming a MedDiet pattern, as reflected by the overall low scores of adherence. Second, in early autopsy research, CAC manifestations have been found in individuals younger than 35 years of age [79,80,81,82]. However, research has also shown that sex and age affect the development of CAC, with females showing similar rates to men when they are in their 70s [13]. Prior to this age, women tend to show a lower risk of CAC and a lower severity of CAC [13]. Because our study included young adults at baseline (mean ages of 37 y and 39 y in controls and T1D, respectively) and contains slightly more females, it may be too early to observe CAC in this group; 32% had a CAC > 0 at baseline. Additionally, research has shown that pericardial fat is positively correlated with CAC, and PAT may exert proatherogenic and proinflammatory effects on the nearby coronary vasculature, thus contributing to advanced CVD pathologies [83,84,85,86,87]. This could explain why we observed significant associations with PAT rather than CAC as an advanced outcome in our study.

The strengths of our study include the large sample size and the inclusion of individuals with and without T1D. To our knowledge, this is the first study to assess the associations of MSDPS with CAC and PAT in adults with and without diabetes in the same cohort. The MSDPS has many strengths, including using the Mediterranean diet pyramid to construct its point system and point deductions for overconsumption [35]. Additionally, the MSDPS accounted for the consumption of non-Mediterranean foods in its scoring, which has not been included in other MedDiet scoring patterns [35]. Lastly, this study measured PAT as a risk factor for CAC, which has not been reported in other studies.

While this study has many strengths, it also has some limitations. The case-control study design may limit the generalizability to other populations and is not able to address causality. Secondly, the self-reporting of dietary components is subject to recall bias, as individuals may inflate or reduce the reporting of food groups that they perceive as healthy or unhealthy. Additionally, the FFQ used did not have olive oil as a separate category but included an option to specify other oils used for cooking; no olive oil use was documented, which likely affected our sample’s adherence scores. There is also the possibility of residual confounding, although we included all relevant covariates in our models. Finally, based on our previous publications of secondary aims using data from the primary study [42,88,89,90,91], we did not perform a separate power calculation for this analysis.

In conclusion, the baseline data from the CACTI study show that a greater adherence to a MedDiet is associated with a lower volume of pericardial adipose tissue in non-diabetic controls only, but not with coronary artery calcification in patients with or without diabetes. Higher PAT volumes have been correlated with CAC development and, thus, coronary artery disease in adults. Therefore, our observed associations may be relevant in the early management of CAC. Overall, Mediterranean-style dietary recommendations must be included in the medical nutrition therapy for the prevention and management of CVD in US adults. Future research must examine these associations in multiple years of follow-up as well as in adults with other chronic diseases leading to vascular dysfunction.

## Figures and Tables

**Table 1:** Baseline characteristics of participants.

Variables	Non-Diabetic Controls (*n* = 692; % = 55.14)		Type 1 Diabetes (*n* = 563; % = 44.86)		*p*-Value
	Mean	SD	Mean	SD	
Age, years	39	9	37	9	**<0.0001**
BMI, kg/m^2^	26	5	26	4	0.9089
Calories, kcal/day	1821	619	1768	613	0.1305
HDL-C, mg/dL	50.6	14.5	56.3	16.3	**<0.0001**
LDL, mg/dL	115	33	101	29	**<0.0001**
HbA1c, %	5.5	0.4	7.9	1.2	**<0.0001**
Systolic blood pressure, mm Hg	114	12	117	14	**<0.0001**
PAT (cm^3^)	35.3	23.5	30.5	16.6	**<0.0001**
MSDPS total and component scores	**Score (Servings per Day)**	**SD**	**Score (Servings per Day)**	**SD**	
Overall MSDPS	25.4	7.8	25.0	7.8	0.4042
Whole grains	1.96 (1.63)	1.8 (2.2)	2.29 (2.14)	1.9 (3.4)	**0.0013**
Fruits	4.47 (2.16)	3.0 (3.6)	4.51 (2.61)	3.0 (4.2)	0.8303
Vegetables	3.81 (2.97)	2.3 (5.5)	3.89 (3.84)	2.4 (9.0)	0.5316
Dairy	5.50 (2.06)	3.0 (2.6)	5.52 (2.43)	3.1 (3.2)	0.8907
Wine	0.89 (0.26)	1.6 (0.9)	0.71 (0.20)	1.6 (0.8)	**0.0484**
Fish	2.68 (0.39)	2.3 (1.2)	2.27 (0.32)	2.2 (1.2)	**0.0013**
Poultry	5.18 (0.63)	3.0 (1.1)	5.02 (0.58)	3.0 (1.1)	0.3598
Legumes	3.89 (0.55)	3.0 (1.0)	3.67 (0.66)	3.0 (1.7)	0.1938
Potatoes	4.58 (0.41)	2.6 (0.7)	4.59 (0.49)	2.7 (1.1)	0.9441
Eggs	4.38 (0.30)	3.8 (0.5)	4.33 (0.31)	3.9 (0.39)	0.8262
Sweets	0.10 (5.23)	0.9 (5.9)	0.07 (5.59)	0.6 (7.6)	0.5223
Meat	0.65 (0.98)	2.0 (1.5)	0.69 (1.18)	2.1 (2.0)	0.7288
	**Count**	**%**	**Count**	**%**	
Sex (female)	349	50	319	57	**0.0359**
Hispanic	59	9	15	3	**<0.0001**
NHW	582	84	536	95	**<0.0001**
CAC > 0	179	26	223	40	**<0.0001**
	**Median**	**IQR**	**Median**	**IQR**	
Triglycerides, mg/dL	103	(75–154)	78	(62–108)	**<0.0001**
Physical activity, min/week	84	(0–300)	42.5	(0–300)	0.3529

*p* < 0.05 in bold font. Abbreviations: SD = Standard Deviation; MSDPS = Mediterranean-Style Dietary Pattern Score; NHW = Non-Hispanic White; BMI = Body Mass Index; HDL-C = High-Density Lipoprotein Cholesterol; LDL = Low-Density Lipoprotein; SBP = Systolic Blood Pressure; CAC = Coronary Artery Calcification; PAT = Pericardial Adiposity.

**Table 2:** Baseline associations of Mediterranean-Style Dietary Pattern Score with the presence of Coronary Artery Calcification.

Coronary Artery Calcification > 0						
Variable + Model	Pooled Analysis		Non-Diabetic Controls		T1D	
	OR	95% CI	OR	95% CI	OR	95% CI
MSDPS + Model 1 ^a,d^	1.00	(0.98, 1.02)	0.995	(0.97, 1.02)	1.00	(0.98, 1.03)
MSDPS + Model 2 ^b,d^	1.00	(0.98, 1.02)	1.01	(0.98, 1.03)	1.00	(0.97, 1.03)
MSDPS + Model 3 ^c,d^	1.00	(0.98, 1.02)	1.01	(0.98, 1.04)	1.00	(0.97, 1.03)

^a^ Adjusted for age, sex, and diabetes. ^b^ Adjusted for age, sex, diabetes, total calories, HbA1c, and BMI. ^c^ Adjusted for age, sex, diabetes, total calories, BMI, HDL-C, LDL, and SBP. ^d^ In analyses stratified by diabetes status, diabetes was removed from the models. Abbreviations: MSDPS = Mediterranean-Style Dietary Pattern Score; CAC = Coronary Artery Calcification; OR = Odds Ratio; CI = Confidence Interval; BMI = Body Mass Index; HDL-C = High-Density Lipoprotein Cholesterol; LDL = Low-Density Lipoprotein; SBP = Systolic Blood Pressure; T1D = Type 1 Diabetes.

**Table 3:** Baseline associations of Mediterranean-Style Dietary Pattern Score with the volume of Pericardial Adiposity.

Pericardial Adiposity ^†^						
Variable + Model	Pooled Analysis		Non-Diabetic Controls		T1D	
	Estimate	95% CI	Estimate	95% CI	Estimate	95% CI
MSDPS + Model 1 ^a,e^	**−0.005**	**(−0.008, −0.001)**	**−0.007**	**(−0.012, −0.003)**	−0.001	(−0.006, 0.004)
MSDPS + Model 2 ^b,e^	**−0.005**	**(−0.01, 0.00)**	**−0.007**	**(−0.011, −0.002)**	−0.0008	(−0.006, 0.004)
MSDPS + Model 3 ^c,e^	**−0.003**	**(−0.006, −0.0004)**	**−0.005**	**(−0.009, −0.001)**	−0.001	(−0.005, 0.003)
MSDPS + Model 4 ^d,e^	−0.002	(−0.006, 0.001)	**−0.005**	**(−0.009, −0.0001)**	0.002	(−0.003, 0.006)

Values in bold indicate *p* < 0.05. ^a^ Adjusted for age, sex, and diabetes status. ^b^ Adjusted for age, sex, diabetes status, HbA1c, and total calories. ^c^ Adjusted for age, sex, diabetes status, and BMI. ^d^ Adjusted for age, sex, diabetes status, LDL, triglycerides, and physical activity. ^e^ In analyses stratified by diabetes status, diabetes was removed from the models. ^†^ log-transformed. Abbreviations: MSDPS = Mediterranean-Style Dietary Pattern Score; PAT = Pericardial Adiposity; CI = Confidence Interval; BMI = Body Mass Index; LDL = Low-Density Lipoprotein; T1D = Type 1 Diabetes.

**Table 4:** Age, sex, diabetes status, HbA1c, and calories-adjusted analyses of the associations of Mediterranean-Style Dietary Pattern Score components with Coronary Artery Calcification presence and Pericardial Adiposity volume.

MSDPS Component Scores/Day	CAC > 0			PAT ^†^		
	OR	95% CI	*p*-Value	Parameter Estimate	95% CI	*p*-Value
Whole grains	0.94	(0.87, 1.02)	0.132	−0.01	(−0.03, 0.00)	0.102
Fruits	0.96	(0.91, 1.00)	0.054	**−0.02**	**(−0.03, −0.01)**	**<0.0001**
Vegetables	0.98	(0.92, 1.04)	0.514	−0.01	(−0.02, 0.00)	0.084
Dairy	1.00	(0.96, 1.05)	0.955	−0.004	(−0.013, 0.004)	0.355
Wine	0.96	(0.88, 1.04)	0.333	**−0.03**	**(−0.05, −0.02)**	**0.0001**
Fish	1.00	(0.94, 1.07)	0.957	−0.01	(−0.02, 0.00)	0.149
Poultry	1.03	(0.98, 1.07)	0.273	0.01	(−0.0007, 0.017)	0.072
Legumes	0.97	(0.92, 1.01)	0.132	0.002	(−0.01, 0.01)	0.680
Potatoes	1.02	(0.97, 1.08)	0.355	0.01	(−0.002, 0.02)	0.117
Eggs	1.02	(0.98, 1.05)	0.305	0.001	(−0.01, 0.01)	0.818
Sweets	1.17	(0.97, 1.40)	0.096	−0.002	(−0.04, 0.03)	0.895
Meat	0.95	(0.89, 1.03)	0.229	**−0.03**	**(−0.04, −0.01)**	**<0.0001**

^†^ Log-transformed. *p* < 0.05 in bold font. Abbreviations: MSDPS = Mediterranean-Style Dietary Pattern Score; CAC = Coronary Artery Calcification; PAT = Pericardial Adiposity; OR = Odds Ratio; PE = Parameter Estimate; CI = Confidence Intervals.

## Data Availability

The datasets analyzed in the current study are not publicly available due to ethical reasons and because our participants only gave their consent for the use of their data by the original team of investigators.
